# Evaluation of Eosinophil-to-Lymphocyte Ratio and Eosinophil Count as Predictive Markers in Fibromyalgia Syndrome

**DOI:** 10.12669/pjms.41.7.12064

**Published:** 2025-07

**Authors:** Buyukavci Raikan, Kaya Caglar Ilhan, Yilmaz Ramazan

**Affiliations:** 1 Buyukavci Raikan, Physical Medicine and Rehabilitation Clinic, Konya Beyhekim Training and Research Hospital, University of Health Sciences, Devlethane Street No:2/A, 42060, Selcuklu, Konya, Turkey; 2 Kaya Caglar Ilhan, Department of Physical Medicine and Rehabilitation, Konya Beyhekim Training and Research Hospital, University of Health Sciences, Devlethane Street No:2/A, 42060, Selcuklu, Konya, Turkey; 3 Yilmaz Ramazan, Department of Physical Medicine and Rehabilitation, Konya Beyhekim Training and Research Hospital, University of Health Sciences, Devlethane Street No:2/A, 42060, Selcuklu, Konya, Turkey

**Keywords:** Fibromyalgia syndrome, Eosinophil-to-lymphocyte ratio, Inflammation, Hemogram

## Abstract

**Objective:**

This study aimed to investigate the predictive role of eosinophil-to-lymphocyte ratio (ELR), and eosinophil count (EC) in patients with Fibromyalgia syndrome (FMS) and to compare with healthy controls.

**Method:**

This retrospective observational study was conducted from January 1^st^ to December 31st, 2024 in Konya Beyhekim Training and Research Hospital. 109 FMS (101 female/8 male) patients who met the 2016 American College of Rheumatology (ACR) classification criteria and 85 (75 female/10 male) healthy controls were included in the study. Demographic characteristics, neutrophil, lymphocyte, and eosinophil counts (EC) levels were recorded. ELR, neutrophil-lymphocyte ratio (NLR) calculated.

**Results:**

In patients with FMS, the blood ELR, NLR, and EC were significantly higher compared to the control group (p<0.05). In the roc curve analysis, blood NLR had 62.4% sensitivity and 50% specificity; blood ELR and EC ≥had 60.6% sensitivity but < 40 % specificity in predicting fibromyalgia.

**Conclusion:**

The results of this study showed that ELR and EC are promising inflammatory markers in the diagnosis of fibromyalgia and should be evaluated in a larger population along with other hemogram parameters.

## INTRODUCTION

Fibromyalgia syndrome is defined as a syndrome characterized by chronic widespread body pain, fatigue, sleep disturbance, and cognitive impairment, and many causes have been implicated in its etiopathogenesis. The interaction of various mechanisms, including genetic predisposition, stress, peripheral (inflammatory) and central (cognitive-emotional) mechanisms, is thought to lead to neuromorphological changes (nosiplastic pain) and impaired pain perception.^1^ The diagnosis of fibromyalgia syndrome is still made according to the ACR 2016 criteria, and there is no definitive laboratory parameter that can support the diagnosis or be used in treatment follow-up.^2^ Although the incidence is reported to be 2-4% in the general population, overdiagnosis, misdiagnosis, and underdiagnosis are frequently observed.^3^ Therefore, studies that can provide laboratory support for diagnosis and follow-up are emphasized.

Routine blood tests are the most accessible clinical tools and have been shown to reflect the progression of various diseases by quantifying the counts and ratios of inflammatory cells in peripheral blood across a wide spectrum.

It has been reported that certain inflammatory protein levels are higher in the plasma and cerebrospinal fluid of FMS patients, indicating systemic and neuroinflammation.^4^ Total leukocyte count and subtypes and their ratios (neutrophil count; platelet count; EC: eosinophil count; ELR: eosinophil-to-lymphocyte ratio; NLR: neutrophil-to-lymphocyte ratio; PLR: platelet-to-lymphocyte ratio) have recently been shown in many studies to be indicators of chronic inflammation.^5^-^7^ During an inflammatory response, the ratio of circulating leukocytes changes. Neutrophilia is associated with relative lymphopenia. In the literature, NLR and PLR have been suggested to have prognostic significance in cardiovascular disease, diabetes mellitus, hypertension, cirrhosis, familial Mediterranean fever and malignancies.

NLR has also been shown to be a prognostic and predictive marker in osteoarthritis and FMS.^8^,^9^ Eosinophils are primarily tissue-resident cells. In the bone marrow, they differentiate from stem cell-derived CD34+ multipotential myeloid progenitors in response to a variety of T cell-derived eosinophilia cytokines and growth factors, including interleukin (IL)-3, granulocyte-macrophage (GM)-CSF, and IL-5. Eosinophils tend to preferentially localize to specific tissues and organs exposed to the external environment, particularly the submucosal and loose connective tissues of the skin, gastrointestinal tract, genital tract, and lungs.^10^ The percentage of eosinophils in peripheral blood is 3-5% and their absolute number is 350-500/µL. Eosinophilia is usually associated with parasitic helminth infections, allergic diseases, and various disease processes, with increased production of eosinophils and accumulation of blood and tissue eosinophils.^10^ It has also been shown that eosinophil levels can be used as an indicator of inflammation.^11^

This study hypothesizes that hematological inflammatory markers such as EC and ELR are significantly different in patients with fibromyalgia compared to healthy controls. Limited research exists examining these markers in FMS, which underscores the novelty and necessity of this investigation. This study aimed to investigate the predictive role of NLR, ELR ratios, and EC in patients with FMS and to compare them with healthy controls.

## METHODS

This retrospective observational study was conducted from January 1st to December 31st, 2024 in Konya Beyhekim Training and Research Hospital. All patients included in the study met the 2016 ACR classification criteria for FMS. Patients were excluded if they had a diagnosis of chronic inflammatory disease, acute or subacute infection, hypertension, hypercholesterolemia or diabetes, psychiatric or neurologic disease, malignancy, known thrombotic or bleeding disorder or anticoagulant therapy, or allergic rhinitis/asthma. Of the 304 patients with a screening diagnosis of FMS, 109 FMS patients and 85 healthy controls were included in the study after meeting the exclusion criteria. Demographic data and complete blood count parameters (count of leukocytes, neutrophils, eosinophils, monocytes, and basophils; mean platelet volüme (MPV); platelet distribution width (PDW), Systemic Inflammation Index (SII), Systemic Inflammation Response Index (SIRI)) of all participants were recorded. NLR, MLR, ELR, BLR, SII, and SIRI were calculated according to the following equations:

NLR= neutrophil count (10^9^/L)/ lymphocyte count (10^9^/L)

MLR= monocyte count (10^9^/L)/ lymphocyte count (10^9^/L)

ELR= eosinophil count (10^9^/L)/ lymphocyte count (10^9^/L)

BLR= basophil count (10^9^/L)/ lymphocyte count (10^9^/L)

SII = platelets x (neutrophils/lymphocytes)

SIRI = neutrophil x (monocyte/lymphocyte)

All parameters were measured in venous blood collected from each patient using an automatic hematology analyzer-F800 (Hamburg, Germany).

### Ethical Approval:

Approval of study was taken from Necmettin Erbakan University Health Sciences Scientific Research Ethics Committee (Approval no: 2025/921 Date January 1^st^ 2025). Permission was also obtained from institution from where the data were collected. It was conducted by the principles of the Declaration of Helsinki.

### Statistical Analysis:

Statistical analysis were performed using the Statistical Package for the Social Sciences (SPSS) for Windows, version 22.0 (SPSS Inc., Chicago, IL, USA). The normality of distribution for continuous variables was assessed using the Kolmogorov-Smirnov test. Normally distributed data were expressed as mean ± standard deviation (SD), whereas non-normally distributed data were presented as median (minimum–maximum). Comparisons between groups were performed using the independent samples t-test for normally distributed variables, and the Mann-Whitney U test for non-normally distributed variables.

To evaluate the diagnostic performance of hematological parameters, the Receiver Operating Characteristic (ROC) curve analysis was performed. For each parameter, the area under the ROC curve (AUC), sensitivity, specificity, and optimal cut-off points were calculated. The cut-off values were determined using the Youden Index. Statistical significance was considered at p < 0.05. AUC values were reported with 95% confidence intervals (CI).

## RESULTS

Total 101 of 109 FMS patients were female with a mean age of 49.10±9.08 years; 75 of 85 healthy controls were female with a mean age of 46.61±11.76 years; there was no statistical difference in age and gender distribution between the two groups (p>0.05). Total leukocyte count and eosinophil count were statistically significantly higher in fibromyalgia patients than in healthy controls (p=0.002 and p<0.001, respectively). Similarly, NLR, ELR, PDW, SII, and SIRI values were found to be higher in the FMS group. Specifically, mean ELR, EC, and NLR values were 0.066 ± 0.051, 0.14 ± 0.09, and 1.94 ± 0.69 in the FMS group, respectively, compared to 0.048 ± 0.037, 0.10 ± 0.07, and 1.71 ± 0.64 in the control group (Table-I).

**Table-I:** Comparison of laboratory values between patient and control groups.

	Patients with FMS (n:109)	Healthy control (n:85)	P value
Age	49.10±9.08	46.61±11.76	0.09
Sex			
Female	101	75	0.29^b^
Male	8	10	
WBC	6.99±1.49	6.30±1.57	0.002
EC	0.14±0.09	0.10±0.07	<0.001
NLR	1.94±0.69	1.71±0.64	0.01
ELR	0.066±0.051	0.048±0.037	0.007
MLR	0.21±0.08	0.20±0.06	0.13
BLR	0.013±0.011	0.013±0.022	0.96
MPV	10.80±1.06	10.85±1.03	0.74
PDW	13.59±1.81	13±1.36	0.01
SII	63.36±29.37	53.39±19.58	0.008
SIRI	0.92±0.41	0.73±0.38	0.001

WBC: white blood cell; EC: eosinophil count; NLR: neutrophil-lymphocyte ratio; ELR: eosinophil-lymphocyte ratio; MLR: monocyte-lymphocyte ratio; BLR: basophil-lymphocyte ratio; MPV: mean platelet volume; PDW: platelet distribution width, SII: Systemic Inflammation Index, SIRI: Systemic Inflammation Response Index. P^b:^ Mann-Whitney U Test.

**Fig.1:**
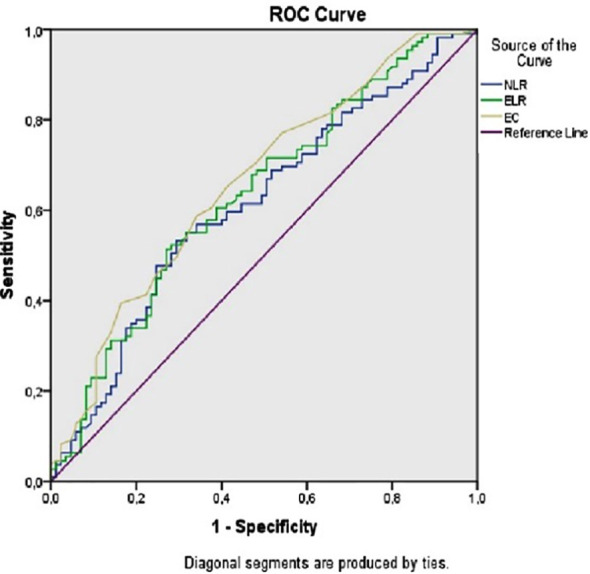
ROC curve NLR; ELR and EC of patients with FMS. *NLR: neutrophil-lymphocyte ration; ELR: eosinophillymphocyte ratio; EC: eosinophil count; FMS: fibromyalgia syndrome*.

The ROC curve can be used to investigate the relationship between NLR, ELR, EC, and FMS in hemogram values (Fig.1). The area under the curve (AUC) for NLR was 0.612 (95% confidence interval: 0.532-0.692; p=0.008), cut-off 1.62, sensitivity 62.4%, specificity 50% Using a cut-off value of 1.62, admission blood NLR predicted fibromyalgia with 62.4% sensitivity and 50% specificity. For ELR, AUC was 0.635 (95% CI: 0.556–0.713; p=0.001), cut-off 0.046, sensitivity 60.6%, specificity 38.8%. For EC, AUC was 0.661 (95% CI: 0.532–0.692; p<0.0001), cut-off 0.105, sensitivity 60.6%, specificity 37.6% (Table-II).

**Table-II:** ROC analysis results.

	AUC (95% CI)	Cutt-off	p value	Sensitivity (%)	Specificity (%)
NLR	,612 (0.532-0.692)	1.62	0.008	62.4	50
ELR	,635 (0.556-0.713)	0.046	0.001	60.6	38.8
EC	,661 (0.532-0.692)	0.105	0.0001	60.6	37.6

AUC: Area under curve; CI: Confidence Interval; NLR: neutrophil-lymphocyte ratio; ELR: eosinophil-lymphocyte ratio: EC: eosinophil count; p value < 0.05.

***Note:*** ROC curve analysis was used for each parameter. Area under the curve (AUC) values were calculated, and significance was determined at p<0.05. Cut-off values were selected based on the Youden Index.

## DISCUSSION

In this study, eosinophil-to-lymphocyte ratio, eosinophil count, and other complete blood count parameters were evaluated in fibromyalgia patients. ELR, EC, NLR, PDW, SII, and SIRI were found to be significantly higher in fibromyalgia patients compared to healthy controls. The relationship between inflammation and markers that can be detected with inexpensive laboratory tests in FMS patients will shed new light on the etiopathogenesis and treatment of the disease. Nosiplastic pain is defined by the IASP as a third category of pain that cannot be characterized by nociceptor activation or neuropathy without clear evidence of actual or threatened tissue damage causing the pain.^12^ Under this definition, FMS remains a disorder characterized by chronic widespread pain, the pathogenesis of which is not fully understood and which affects a significant proportion of the world’s population.^13^ Studies on changes in hemogram parameters used for diagnosis and follow-up have been widely reported in the literature in recent years.^14^

In a study conducted in patients with FMS, NLR and PDW were found to be promising inflammatory markers indicative of fibromyalgia.^8^ Al-Nimer et al. found an association between NLR levels and disease activity in FMS.^4^ On the other hand, in pain classifications such as FMS, headache, and migraine are also included in the nosiplastic pain category.^15^ A recent study of changes in hematologic and inflammatory markers during the non-attack period in migraineurs without aura supports that hemoconcentration and chronic inflammation persist even in the absence of pain attacks.^16^ The fact that these hematologic marker changes found in nonattachment pain categories are related to the inflammatory response is instructive for comprehensive studies.

Leukocyte counts and/or their ratios are often chosen as a measure to assess the presence of inflammation because of their affordability and convenience. Blood eosinophils are an important component of the immune system and play a role in regulating over-activation of immune function and promoting inflammatory responses.^17^ Chemokines and cytokines all stimulate eosinophil accumulation, while eosinophil activation can release cytokines to further promote the inflammatory cascade response. Previous studies have demonstrated the predictive role of ELR in various diseases. 18 ELR has been included in studies as a useful parameter in predicting disease and evaluating response to treatment, particularly in asthma, COPD, and atopic dermatitis.^19^,^20^

It has been found that peripheral blood eosinophilia is often associated with cardiac involvement and cardiovascular complications; similarly, it has been found to be associated with chronic kidney disease and these have been found to increase patient mortality.^21^ Kargili A et.al concluded that eosinophilia could be observed in various rheumatologic conditions in a group of one thousand patients diagnosed with rheumatic diseases, but eosinophilia due to corticosteroid use was more common than reported in the patients included in the study. In the same study, the rate of eosinophilia in patients with FMS was found to be 10.7%.^10^

In a recent study, eosinophil count and ELR were found to be new predictive markers for osteoarthritis.^22^ Eosinophils are highly specialized hematopoietic effector cells that produce, accumulate, and secrete various bioactive compounds. Under various physiological and pathological conditions, eosinophils migrate to their target organs to release their products, thereby promoting local inflammation and even causing tissue damage. However, this study did not find an association between NLR and OA.^23^ Although the exact involvement of NLR in the process is still unclear, it should be recognized that numerous studies have provided evidence for the critical role of neutrophils in the pathophysiology of OA.^9^,^23^

Recent studies have demonstrated the clinical utility of novel inflammatory indices such as the SII and the SIRI in various chronic inflammatory and autoimmune conditions, including rheumatoid arthritis, systemic lupus erythematosus, and even cancer-related fatigue. 24,25 These composite markers, which integrate multiple hematologic parameters (e.g., neutrophils, lymphocytes, monocytes, and platelets), provide a broader reflection of the immune-inflammatory balance than individual cell counts or simple ratios. In our study, both SII and SIRI were found to be significantly higher in patients with fibromyalgia compared to healthy controls, further supporting the growing body of evidence suggesting that systemic low-grade inflammation plays a role in FMS pathogenesis. Given their accessibility, cost-effectiveness, and ability to capture complex inflammatory dynamics, these indices may hold promise as objective biomarkers for disease activity, symptom burden, or treatment response in future fibromyalgia research.

Despite the statistical significance of these markers, the specificity values observed in our ROC analysis were relatively low (all below 50%), which limits their standalone diagnostic use. This suggests that while these markers can be useful screening or supportive tools, they should not be used alone to confirm the diagnosis of FMS. The overlap in inflammatory patterns among other chronic conditions may partly explain this limitation. Future research integrating these indices with other clinical or biochemical parameters may improve diagnostic precision.

This study adds new data to the existing literature by evaluating EC and ELR as potential inflammatory markers in FMS. The clinical relevance lies in the potential use of these easily accessible and low-cost markers to support diagnosis and monitor inflammatory activity in patients. The major strengths of this study are the control group and the high number of patients. However, further studies are needed to confirm these findings in different populations, investigate possible correlations with symptom severity or treatment response, and elucidate the pathophysiology of FMS.

### Limitations:

It was performed in a single center with a retrospective design. Furthermore, inflammatory parameters were not associated with parameters such as disease activity and quality of life. Future multicenter, prospective studies with larger data sets are needed to confirm these findings.

Another limitation of this study is the gender imbalance, as the vast majority of participants were female. While this reflects the known epidemiological distribution of FMS, it restricts the generalizability of the findings to male patients and underscores the need for more balanced future cohorts.

## CONCLUSION

The results of this study showed that ELR and EC are promising inflammatory markers in the diagnosis of fibromyalgia and should be evaluated in a larger population along with other hemogram parameters.

### Author’s Contributions:

**BR:** Data curation, methodology, formal analysis, preparation of original draft.

**KCI:** Study concept, data curation, Critical review.

**YR:** Study concept, formal analysis, Critical review.

All authors have approved the final version and are responsible and accountable for the accuracy and integrity of the work.
